# Increased Plasma Levels of Mitochondrial DNA and Normal Inflammasome Gene Expression in Monocytes Characterize Patients With Septic Shock Due to Multidrug Resistant Bacteria

**DOI:** 10.3389/fimmu.2020.00768

**Published:** 2020-05-05

**Authors:** Stefano Busani, Sara De Biasi, Milena Nasi, Annamaria Paolini, Sophie Venturelli, Martina Tosi, Massimo Girardis, Andrea Cossarizza

**Affiliations:** ^1^Intensive Care Unit, Policlinico di Modena, University of Modena and Reggio Emilia, Modena, Italy; ^2^Department of Medical and Surgical Sciences for Children and Adults, University of Modena and Reggio Emilia, Modena, Italy; ^3^Department of Surgical, Medical and Dental Morphological Sciences Related to Transplant, Oncology and Regenerative Medicine, University of Modena and Reggio Emilia, Modena, Italy; ^4^National Institute for Cardiovascular Research – INRC, Bologna, Italy

**Keywords:** septic shock, circulating mtDNA, multidrug resistance bacteria, inflammasome, intensive care unit

## Abstract

**Introduction:** The activity and regulation of inflammasome is receiving increasing attention in septic shock. Moreover, there is a growing body of evidence suggesting that mitochondrial DNA (mtDNA) can play a role as biomarker of disease severity and even mortality both in adults and children in critically ill setting. However, no data are available on the amount of circulating mtDNA and inflammasome gene expression in multi-drug resistant (MDR) bacteria septic shock. For this reason, the aim of this study was to determine whether plasma mtDNA levels and inflammasome gene expression in monocytes could be related to severity in patients admitted to intensive care unit (ICU) with septic shock due to MDR pathogens.

**Materials and Methods:** Peripheral blood mononuclear cells (PBMC) and plasma were isolated from up to 20 ml of venous blood by density gradient centrifugation in patients admitted to ICU with the diagnosis of septic shock due to MDR-bacteria. Then, CD14+ monocytes were sorted, and RNA and DNA were extracted. NLRP3, PYCARD, AIM2 and NAIP expression level was analyzed by RT-PCR. Plasma circulating mtDNA levels were quantified by digital droplet PCR. Basal and outcome characteristics of the patients were collected. Age-matched healthy subjects were chosen as controls.

**Results:** Nineteen patients with septic shock and 20 healthy subjects were enrolled in the study. A small trend toward an increased expression of inflammasome genes was observed in septic shock patients, who also displayed a marked tendency to an increased expression of IL-18 and IL-1β genes. Circulating mtDNA levels were significantly higher in septic shock patients if compared to healthy subjects, and patients who died in ICU were characterized by higher level of mtDNA if compared to those who were dismissed after 7 days. No correlations were found between mtDNA and inflammasome level and other clinical variables.

**Conclusion:** Despite many limitations, our data suggest that in patients with septic shock caused by MDR pathogens the expression of main inflammasome genes was comparable to that of healthy patients without infection. Furthermore, our data evidence a possible role of mtDNA as a prognostic marker of severity in septic shock from MDR.

## Introduction

Septic shock still represents the pathology with the highest risk of mortality worldwide despite the knowledge and attention dedicated to this disease over the past 20 years. Recent investigations of the pathogenic host response to infection have highlighted a different behavior depending on whether there is a hyperinflammatory state or a hypo-reactivity of the immune system (1). These new perspectives have brought up the role of pathogen-associated molecular patterns (PAMPs) and danger-associated molecular patterns (DAMPs) in the initiation and propagation of the inflammatory cascade. This “Danger Model” stipulates that when cells are injured they release their components into the extracellular space, which in turn drives an immune or inflammatory response (2). Inflammasomes, such as NLRP3, PYCARD, AIM2, and NAIP are multimeric protein complexes that serve as important cytosolic pattern recognition receptors required for recognizing DAMPs and PAMPs. The activation of inflammasome signaling pathways is involved in mounting a proinflammatory immune response by regulating the maturation from precursors of IL-1β, IL-18, IL-33, cytokines that can induce pyroptosis. Recently, mitochondrial DAMPs have been identified as important mediators of the innate immune response and implicated in various conditions such as trauma, sepsis, and autoimmune disorders [reviewed in (3)]. Accordingly, the ability of mitochondrial (mt) DNA to act as DAMPs in the activation/inhibition of the inflammatory cascade has been recently investigated (4, 5). In critically ill setting, mainly in septic shock patients, there is a growing body of literature suggesting that mtDNA plasma levels can probably be used as biomarker of disease severity and even mortality both in adults and children (6–8).

The role of inflammasome and mtDNA is a research field that is receiving more and more attention in septic shock, but, to our knowledge, no data exist on these two parameters during multi drug resistant (MDR)-bacteria septic shock. For this reason, we investigated whether plasma mtDNA levels and inflammasome gene expression in monocytes, cells that are crucial for innate immune response, could be related to severity in patients admitted to intensive care unit (ICU) with septic shock due to MDR pathogens infection.

## Materials and Methods

### Patients’ Population and Selection

We performed a prospective observational study in the ICU of the University Hospital Policlinico of Modena (Italy) between April 2014 and December 2018. Evaluation of entry criteria and subsequent enrollment in the study occurred during planned routine patients visits. Inclusion criteria were: patients aged 18 years or older admitted to our ICU with septic shock sustained by documented MDR bacteria. Definitions of septic shock and MDR bacteria are detailed in a previous report (9). Patients with autoimmune or hematologic disease, pregnancy, metastatic cancer, end-stage liver disease, end-of-life decisions, illnesses or with medications known to be toxic to mitochondria were excluded. The type of admission, relevant pre-existing diseases, the primary site of infection, microbiology lab results, SOFA scores when sepsis was diagnosed were collected. Age-matched healthy subjects without infections were chosen as controls (CTR). Blood from patients with septic shock was sampled from the arterial catheter within 24 h from the diagnosis of septic shock. The study was carried out in accordance with recommendations of the Prot. n 2630/CE approved by the Province of Modena Ethical Committee. All subjects gave written informed consent in accordance with the Declaration of Helsinki.

### Biological Sample Collection, Processing and Storage

Peripheral blood mononuclear cells (PBMC) and plasma were isolated from up to 20 ml of venous blood by density gradient centrifugation, using standardized protocols and Lymphoprep reagent from Stemcell (Cambridge, MA, United States). Plasma was then stored at −80°C until use (10). A minimum of 1.5 million CD14+ monocytes were sorted starting from 20 million PBMC through immunomagnetic separation technique (Miltenyi Biotec, Bergish Gladbach, Germany). Purity of monocyte population was always >95%.

### RNA Extraction, Reverse Transcription

RNA was extracted from CD14+ cells through Quick-RNA Miniprep Kit (Zymo Research, Irvine, United States) and quantified using NanoPhotometer NP80 (Implen, Munich, Germany). Then, 20 ng/μl of RNA was reverse transcribed with the iScript cDNA Synthesis kit (Bio-Rad, Hercules, CA, United States).

### Pre-amplification and Real-Time Polymerase Chain Reaction (PCR) for Gene Quantification

In order to obtain a more accurate RNA quantification, cDNA samples were pre-amplified using Sso Advanced PreAmp Supermix (Bio-Rad). Quantification of inflammasome genes was performed by Real-Time PCR as previously described (11). Changes in genes expression between the two groups of patients were calculated through the ΔΔ cycle method.

### DNA Extraction and Digestion

DNA was extracted from plasma samples using QIAmp DNA Mini Kit (QIAGEN, Venlo, Netherlands) and then digested with *Bam*HI enzyme. The reaction mix was prepared as follows: 1 μl *Bam*HI enzyme, 1 μl Buffer Fast Digest, 6 μl DNA, in a total volume of 10 μl. Samples were then incubated in C1000 Touch Thermal Cycler (Bio-Rad) for 5 min at 37°C followed by 5 min at 80°C.

### Total Quantification of mtDNA Circulating in Plasma Using Droplet Digital PCR

Quantitative real-time PCR (qPCR) is often used for the detection of nucleic acid in research and diagnostic, but the methodology has several limitations, first of all the need of preparing dedicated standard curves. Thus, we have used droplet digital PCR (ddPCR) to quantify circulating mtDNA because there is no need of a standard curve, and because the results are less dependent from the efficiency of the reaction. The sample is indeed partitioned in droplets and each of them represents an isolated end-point PCR reaction. The frequency of positive to negative droplets in the reaction mixture thus allows a precise quantification of the concentration of target nucleic acids (12).

Before performing ddPCR, samples were diluted 1:10 in order to obtain more accurate results. Two different assays were performed in this set of experiments: ddPCR assay EIF2C1 (UniqueAssayID: dHsaCP2500349, Bio-Rad) (HEX fluorescence) for genomic DNA and ddPCR assay MT-ND4 for mtDNA (UniqueAssayID:dHsaCPE5043566, Bio-Rad) (FAM, fluorescence). Droplet Digital PCR was performed as previously described (12). Manufacturer’s thermal cycling protocol was optimized, changing the annealing/extension step temperature from 55 to 57°C.

### Statistical Analysis

Categorical (sex) and quantitative (age) variables were compared between groups by χ2 and Mann–Whitney tests, respectively. Differences between controls and septic shock patients were explored with Mann–Whitney test. A *P*-value < 0.05 was considered statistically significant. Data shown in graphs are represented as the mean ± SEM. Statistical analyses were performed using Prism 8.0 (GraphPad Software Inc., San Diego, CA, United States).

## Results

### Patients Characteristics

A total of 19 ICU patients with septic shock induced by MDR pathogens were enrolled. Twenty healthy subjects without infections (8 males/12 females, mean age ± SD, 60.8 ± 4.4 years), were chosen as CTR. ICU patients had septic shock caused mainly by peritonitis and blood stream infection. While multi-resistant Gram-negative bacteria were the most represented pathogens, of these four were *Escherichia Coli* and two each *Pseudomonas Aeruginosa* and *Enterobacter Cloacae* (Table 1). Median SOFA score at ICU admission was 11 with mainly respiratory cardiovascular and hematological dysfunctions. In patients with septic shock the 30-day, ICU and 1-year mortality were 42.1, 52.6, and 63.2%, respectively (Table 1).

**TABLE 1:** Clinical characteristics of the 19 patients with septic shock.

Sex	Male *n* = 12 (63.2%) Females *n* = 7 (36.8%)
Age [years, median (range)]	67 (33–81)
SOFA score [median (range)]	11 (7–21)
ICU mortality *n* (%)	10 (52.6%)
30-day mortality *n* (%)	8 (42.1%)
1-year mortality *n* (%)	12 (63.2%)
Sepsis focus*	
Abdomen *n* (%)	9 (47.4%)
Lung *n* (%)	4 (21.1%)
Blood *n* (%)	9 (47.4%)
Urinary tract *n* (%)	1 (5.3%)
Other *n* (%)	2 (10.5%)
Pathogens	
Gram positive *n* (%)	5 (26.3%)
Gram negative *n* (%)	14 (73.7%)

ICU, intensive care unit, SOFA, sequential organ failure assessment. *To notice that sepsis focus was in 6 cases a mixed focus.

### Inflammasome Gene Expression and mtDNA in Septic Shock Patients and CTR

The gene expression profile of the entire inflammasome pathway was evaluated in isolated monocytes from septic patients. Pattern recognition receptors involved in inflammasomes comprise nucleotide-binding oligomerization domain and leucine-rich repeat-containing receptors (NLR) such as NLRP3 and NAIP, as well as absent in melanoma-2 (AIM-2). Through their caspase activation and recruitment domain (CARD) or pyrin domain (PYD), the inflammasome receptors interact with the adaptor protein ASC, which then recruits pro-caspase-1 *via* its CARD domain and activates the effector caspase through proteolytic cleavage. The activated caspase-1 finally cleaves the immature pro-inflammatory cytokines pro-IL-1β and pro-IL-18.

**FIGURE 1:**
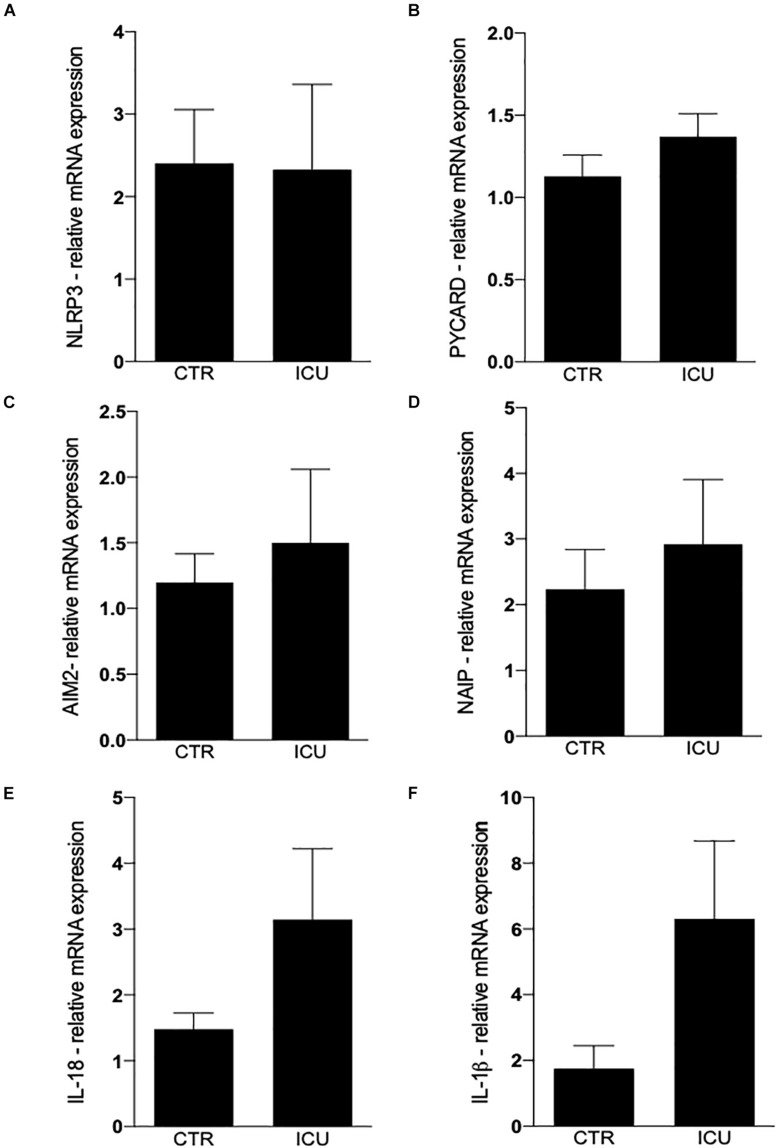
Quantification of inflammasome genes by Real-Time PCR. **(A)** Quantification of NLRP3 in CD14+ cells on CTR and ICU septic shock patients. **(B)** PYCARD, **(C)** AIM2, **(D)** NAIP, **(E)** IL-18, **(F)** IL-1β. Data are represented as mean ± SEM. Analysis was performed using Mann–Whitney test.

The intracellular levels of main inflammasome mRNAs was not significantly different in monocytes from patients of controls, even if a trend toward an increased expression of PYCARD, AIM2 and NAIP was observed in septic shock patients (Figures 1A–D). IL-18 and IL-1β gene expression was higher in patients with septic shock, even if the high variability among patients did not allow to reach statistical significance (Figures 1E,F).

**FIGURE 2:**
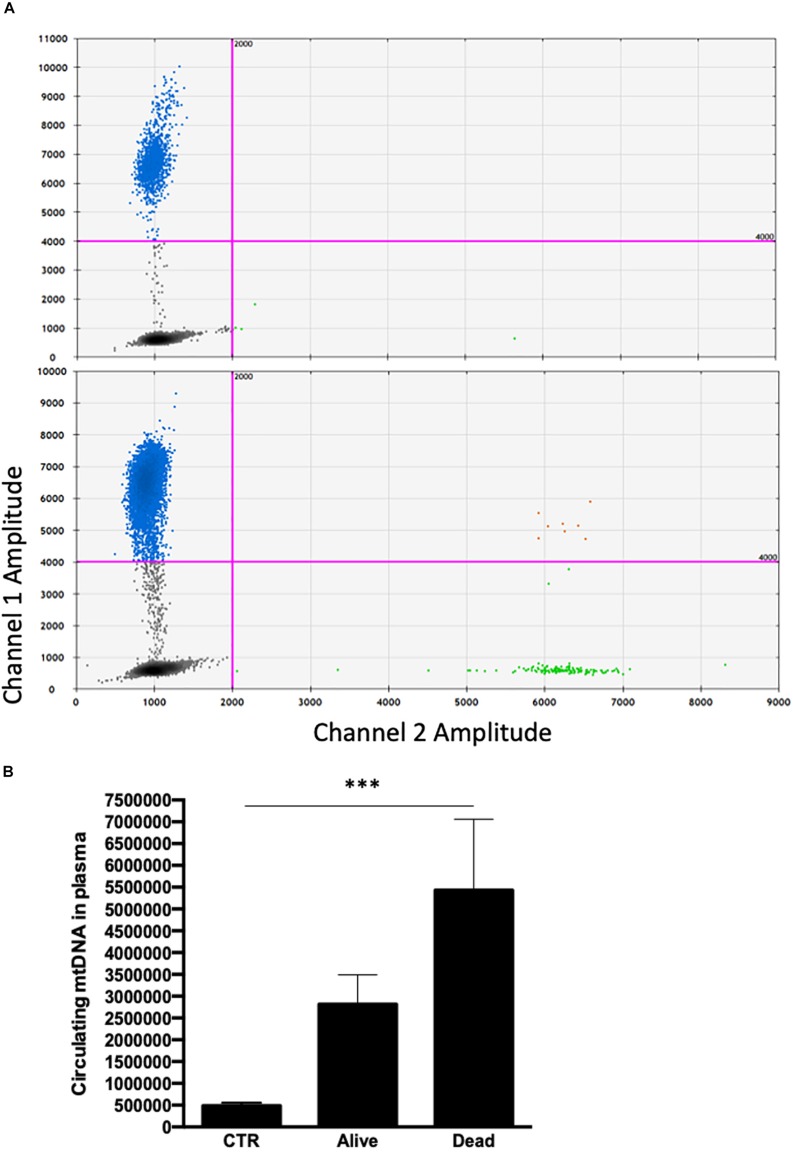
**(A)** Representative 2D scatter plot of a ddPCR result, corresponding to mitochondrial (mt)-ND4 of healthy subject (upper panel) and septic patient (lower panel). The *y* axis shows FAM fluorescence amplitude of the mt-ND4 probe (channel 1) and the *x* axis shows the HEX fluorescence amplitude of the EIF2C1 probe (channel 2). The black cluster represents the negative droplets, the green cluster represents the droplets positive for EIF2C1, the blue cluster represents the droplets positive for mt-ND4 and the orange cluster represents the droplets that are positive for both targets. **(B)** Quantification of mtDNA circulating in plasma on CTR and ICU septic shock patients by ddPCR. Data are represented as mean ± SEM. Analysis was performed using Mann–Whitney test. ***p < 0.05. CTR, controls, ICU, intensive care unit.

Concerning mtDNA, we found that circulating mtDNA levels were significantly higher in septic shock patients if compared to controls (Figures 2A,B), and that patients who died in ICU were characterized by higher level of mtDNA if compared to those patients with septic shock who were then discharged alive from the ICU (Figures 2A,B). No correlations were found between mtDNA and inflammasome gene expression level and other clinical variables reported in Table 1.

## Discussion

The spread of bacteria resistant to many classes of antibiotics is becoming one of the most worrying threats for the scientific community. The search for prognostic biomarkers during septic shock is currently the subject of great debate (13). However, few data exist on the role of inflammatory markers in the prognosis of septic shock caused by MDR bacteria (14) and, in particular, no studies investigate the role of inflammasome and mtDNA in this disease.

Sepsis clearly alters the innate and adaptive immune responses, causing immune suppression, chronic inflammation, and finally exhaustion of several cell defense mechanisms. Understanding how inflammatory processes are orchestrated, and in particular how their complex mechanisms work together could pave the way for the identification not only of suitable therapeutic targets, but also of predictive biomarkers. Here we observed that patients suffering from septic shock due to MDR bacteria, if compared to healthy subjects without infection, are characterized by similar level of inflammasome genes, but significantly higher level of circulating mtDNA.

Circulating cell-free mtDNA is a functional link between cell damage, mitochondrial damage and systemic inflammation and, indeed, mtDNA released after cell death can act as a DAMP, being able to induce an inflammatory response through hypomethylated CpG motifs resembling those of bacterial DNA (15). mtDNA is thus a potent DAMP capable of causing inflammation and propagating an immune response through its interaction with TLR9 and inflammasomes (16).

Plasma circulating mtDNA is elevated in critically ill patients, and increases with age, contributing to the maintenance of the low-grade, chronic inflammation observed in elderly people which has been defined “inflammaging” (17).

Results from clinical trials providing data on mtDNA during sepsis are not conclusive, so it cannot be established if mtDNA is associated with mortality or not (18). This phenomenon could be due to several reasons, that even regard technical aspects such as: (i) the lack of a standardized protocol for the measurements; (ii) the use of plasma or serum (freshly isolated or frozen); (iii) the preparation of plasma with or without the presence of platelets, that contain mitochondria (but not nuclear DNA) and thus can increase the number of molecules of mtDNA (17). To generate this data we took advantage of the sophisticated ddPCR approach, based on partitioning samples in 20,000 or more droplets, each of them representing an isolated end-point PCR reaction. Compared to Real-Time PCR, ddPCR provides absolute nucleic acids quantification and results are less dependent from reaction efficiency. Beyond technical issues, there is also the question of the appropriate patient population. Indeed, it is possible that the mechanism driving mtDNA release differs in patients with cellular injury from sepsis and those with a mechanical injury, as seen in phases like post-trauma or post-surgery. Anyway, in our patients mtDNA was definitely higher in septic shock population, especially in those who died in the ICU.

Concerning the levels of inflammasome gene expression in monocytes between septic shock patients and healthy controls, the hypothesis that MDR patients may have functionally exhausted the inflammatory response agrees with what stated by Hotchkiss et al. (19), who described this kind of patients as an expression of the state of hypo-reactivity of the immune system in the host’s response to infection. Accordingly, our data would confirm that patients suffering from septic shock by MDR pathogens would be in that prevailing phase in which the inflammatory response has exhausted its thrust, returning to the levels of a healthy person not affected by an infectious disease. In other words, we could interpret this as an “abnormal normality,” i.e., as the functional end of a normal inflammatory response that should have been much higher in a clinical situation such as sepsis.

We are aware that it would have been important to study the inflammasome in cells that are capable of triggering various inflammatory pathways, such as macrophages, which typically reside in the tissues. However, we were unable to obtain tissue samples and had to concentrate on circulating monocytes, which are simple enough to obtain, and study gene expression rather than protein levels due to the lack of biological material. In addition to the inability of studying macrophages we must also add that unfortunately, only anecdotical reports (20, 21) have dealt with the role of inflammasomes regarding strains of resistant bacteria, so our hypothesis of “abnormal normality” has to be verified with a more conspicuous number of patients.

The high level of mtDNA in patients with septic shock could also suggest that the exhaustion of inflammasome activity had favored the progression of the sepsis, and thus allowed the onset of further cellular damages.

Our study has two important limiting factors represented by the small size of the sample enrolled and the rather long period of time elapsed between the beginning and the end of the study itself. These limiting factors are related to the difficulty of enrolling patients with septic shock from MDR bacteria with a microbiological tests’ confirmation obtained within 24 h from the onset of the shock (most of our patients had bloodstream infection or secondary or tertiary post-surgical peritonitis). So, our interest was aimed at testing the level of inflammasome gene expression and mtDNA at the early onset of the state of shock.

## Conclusion

In conclusion, even considering the all limitations, our data suggest that patients with septic shock caused by MDR pathogens have a relatively low gene expression of inflammasomes, that is comparable to that of healthy patients without any infection. Furthermore, we show here that mtDNA could be considered an early prognostic marker of severity in septic shock from MDR. Further studies on other, more numerous cohorts are required to confirm our observations.

## Data Availability Statement

The raw data supporting the conclusions of this article will be made available by the authors, without undue reservation, to any qualified researcher.

## Ethics Statement

The studies involving human participants were reviewed and approved by the Comitato Etico Provinciale di Modena e Reggio Emilia. The patients/participants provided their written informed consent to participate in this study.

## Author Contributions

SB and SD designed and carried out the study and drafted the manuscript. SD, MN, and AP performed the plasma tests. SV and MT were involved in clinical data acquisition. MG and AC supervised the study and revised the manuscript.

## Conflict of Interest

The authors declare that the research was conducted in the absence of any commercial or financial relationships that could be construed as a potential conflict of interest.
